# Observational Study on the Preparation of the Implant Site with Piezosurgery vs. Drill: Comparison between the Two Methods in terms of Postoperative Pain, Surgical Times, and Operational Advantages

**DOI:** 10.1155/2019/8483658

**Published:** 2019-09-29

**Authors:** Michele Maglione, Lorenzo Bevilacqua, Federica Dotto, Fulvia Costantinides, Felice Lorusso, Antonio Scarano

**Affiliations:** ^1^Department of Medical Sciences, University of Trieste, 34127 Trieste, Italy; ^2^Department of Medical, Oral and Biotechnological Sciences and CeSi-MeT, University of Chieti-Pescara, Via Dei Vestini 31, 66100 Chieti, Italy; ^3^Department of Oral Implantology, Dental Research Division, College Ingà, UNINGÁ, Cachoeiro de Itapemirim 29312, Brazil

## Abstract

**Purpose:**

Recent advances show that ultrasonic implant site osteotomy is related to a decreased trauma and a better postoperative healing of the surgical site when compared to traditional drilling techniques. The micrometric bone cutting control and the operative advantages related to the piezoelectric approach are also characterized by a learning curve for the clinician in surgical practice and an increased operative duration of the procedure. The aim of this investigation is to compare the operative time, the postoperative pain, and the amount of painkillers taken by the patient during the healing period.

**Methods:**

A total of 65 patients were treated at the Unit of Oral Surgery (Department of Medical Sciences, Surgery and Health, University of Trieste, Italy) using a split mouth model: 75 drill-inserted implants (G1) and 75 piezoelectric device-inserted implants (G2) were placed. The Visual Analogue Scale (VAS) was performed to evaluate the postoperative pain at 15 days from surgery. The operative time and frequency of intake of painkillers were measured.

**Results:**

The G1 and G2 groups showed a significant difference with a higher use of painkillers observed for G1. The G2 patients showed a lower level of pain (VAS) at all experimental times between 8 hours to 7 days (*p* < 0.01) postsurgery. At 15 days, the pain levels were similar for both groups. No differences were found in site preparation duration between the study groups.

**Conclusions:**

The evidence supports the application of the piezoelectric approach compared to the drill's osteotomy as a useful technique for implant site preparation. This trial is registered with NCT03978923.

## 1. Introduction

Piezosurgery has long been applied in implantology for the preparation of implant sites because of its selective cut, cavitational effect, and preservation of soft tissues [1–3]. It achieves the most correct positioning of implants and allows for a more predictable osteointegration while providing an increased respect of bone vitality.

Recent studies have suggested that there are no statistically significant differences in terms of primary stability between implant sites prepared with piezosurgery and those using the traditional technique with dedicated drills [4]. Numerous histological studies conducted both in vitro and in vivo have shown that ultrasonic microvibrations minimize trauma during the cutting action [5]. As a result, bone healing is much faster from both histological and histochemical points of view [6–8].

Furthermore, clinical benefits were highlighted both intraoperatively and postoperatively. The day after surgery, the postoperative edema resulted smaller than those in the sites treated with traditional methods and numerous advantages were found regarding patients' symptoms, both in terms of greater intraoperative comfort and a better postoperative course [9]. Multiple implant rehabilitative protocols involve the use of the piezoelectric technique not only in complex clinical conditions that require, for example, ridge expansion (split crest) or maxillary sinus lift but also in simpler cases, limited only to the preparation of the implant site.

In fact, even in nonadvanced implantology, there are some clinical conditions of objective difficulty regarding the initial stages of surgery. The initial preparation of the implant site using only the pilot drills on the handpiece present in the implant kit, in some circumstances, can be complex; this is due to the fact that the rotation of the cutter, and therefore the macromovements, makes the implant difficult to precisely stabilize at the point established by the operator. In these cases, ultrasound systems are important aids for the surgeon, as a safe, reliable, and advantageous method from an intraoperative (technical-related) point of view.

The main technical and executive advantages of piezoelectric surgery for the operator can be summarized as follows:

- More stable positioning of the guide inserts on the crestal profile for the creation of the first implant site
- Definition of a more correct implant axis that helps in the success of the implant-prosthetic rehabilitation
- Possibility of intraoperative corrections of the implant axis
- Execution of the cortical crestal osteotomy in a more secure way, thanks to the fact that the piezoelectric handpiece is ergonomically pivoted and free from the initial “waving” phenomena typical of rotating systems
- Realization of the initial osteotomy in a less traumatic way and with a greater visibility of the operative field, thanks to the cavitational process with constant irrigation
- Reduction of the emotional impact on the patient, who does not perceive the annoying vibrations caused by the use of drills on the handpiece

The biological advantages, that are however technically related, can be listed as follows:

- Reduction of thermal stress on bone tissue
- Maintenance of a better bone vitality
- Greater respect for osteoblastic turnover and better postresective bone response
- Preservation of the soft tissues and of any noble anatomical structures (inferior alveolar nerve, Schneiderian membrane, etc.) contiguous to the osteotomy [10–12]

Undoubtedly today, ultrasonic surgery techniques are superior to traditional, rotating, or manual instruments, thanks to greater cutting precision and to the possibility of creating more conservative surgical access, as there is no risk of damaging the soft tissues, less operator fatigue, and minimal risk of developing bone thermonecrosis although they present a reduced speed of execution [13–15]. In a previous study, we compared two different implant site preparation techniques using piezoelectric surgery vs. conventional drills with evaluation of pain in patients who were prescribed painkillers, and this research showed less pain in the site prepared with an ultrasonic device [16]. In light of these premises, the main objective of this study was to verify if there were differences between the traditional method with micromotor and dedicated drills and the piezoelectric technique with dedicated tips. The preparation time of the implant site was compared for the two methods, both in absolute terms and according to the type of bone. Then, the operator's learning curve implant survival and the presence of postoperative complications were also evaluated with the piezoelectric method. Finally, attention was paid to the intra- and postoperative pain and to the need for pain medication in the postoperative phases through the use of painkillers.

## 2. Materials and Methods

The study was conducted with the approval of the local ethical committee (n. 88-10.05.2018) of University of Trieste.

The following study included adult patients undergoing implant therapy with insertion of two contralateral conical implants with a diameter between 3.8 and 4.5 mm with a maximum torque of 35 Ncm. In a single sitting, one site was prepared with Ultrasonic device (Esacrom, Imola, Italy), while the contralateral site was prepared with micromotor and dedicated drills. All patients were treated at the Unit of Oral Surgery (Department of Medical Sciences, Surgery and Health, University of Trieste, Italy). Seventy-five patients were enrolled in the study, 44 women and 31 men, aged between 45 and 70, who underwent implant therapy in the period between January 2013 and December 2017. The inclusion criteria were edentulous or partly edentulous with a bilateral loss of teeth in the maxillary or mandible and bone type D2 or D3, according to Misch classification [17]. In general, it is easy to differentiate these bone qualities D2 and D3 than bone types D1 or D4.

The exclusion criteria included general contraindications to implant surgery, severe coagulation disorders, leukocyte or metabolic diseases, immunosuppressed or immunocompromised patients, patients receiving chemotherapy for less than 1 year, patients on therapy or having taken aminobisphosphonates intravenously, patients irradiated at the head or neck, patients with uncontrolled diabetes, pregnant, and lactating patients, patients with poor oral hygiene and motivation, patients needing maxillary sinus lift concomitant with implant insertion, and postextraction sites with acute or purulent infections.

The final sample of the implants inserted was 150 (75 per technique) divided into two groups: the drill-inserted implants (G1) and the ultrasonic device-inserted implants (G2). Each patient subscribed an informed written consent and underwent a preoperative oral hygiene session. Two grams of amoxicillin was administered to each patient in the preoperative phase.

Before surgery, each patient used a 0.2% chlorhexidine mouthwash for one minute.

The surgery was always performed by the same operator (M. M.) to reduce the bias of the study. Locoregional anesthesia was performed with mepivacaine hydrochloride with 1 : 100000 adrenaline.

For the preparation of implant sites using traditional methods, the drills used, following the manufacturer's protocol, were specific for the implant system in use (WINSIX®- BioSAF IN srl, Trezzano Rosa, Trezzano Rosa, Milano, Italy).

For the preparation of the other sites, an ultrasonic device was used (Surgysonic II, Esacrom S.R.L., Imola, Italy). For the final preparation and insertion of implants, 5-6 ultrasound inserts were used in sequence as follows: tip-shape 1st insert (ES012X) and 2nd insert (ES052XG), crown-shape 3rd insert (ES040), 4th insert (ES041), 5th insert (ES043), and 6th insert (ES044).

The implant insertions were performed with a maximum torque of 35 Ncm with manual calibrated torque gauge ratchet. Finally, the cap screws were positioned, and the flaps were sutured with Vycril® 3.0.

Each patient was prescribed a 0.2% chlorhexidine mouthwash to be used twice a day for two weeks and paracetamol 1000 mg (maximum 3 tablets a day) as a pain-relieving therapy.

Each patient included in the study was in possession of two questionnaires, one per technique, for the evaluation of the treatment. In the questionnaires, the patient was asked to trace an “X” representing the level of pain experienced. The questionnaire recorded the individual symptoms experienced during the surgery, after 8 hours, from the 1st to the 7th postoperative day and finally any persistence of the symptoms on the 15th day after surgery. In the same questionnaire, it was also asked to indicate the possible intake of painkillers and the related dose after surgery and in the following six days; moreover, after the fifteenth day, the patient was asked if he would have repeated the experience of the implant surgery. Then, for each patient, a postoperative check was scheduled: after one week, all the patients were recalled for a postoperative control and the removal of the sutures.

For the subjective analysis of the effects of the two methods, it was decided to use the Visual Analogue Scale (VAS). This linear scale is the visual representation of the amplitude of pain that the patient perceives. It is a horizontal line of 100 mm long, in which one end indicates the absence of pain, while the other represents the worst pain imaginable.

During the surgical procedure, the preparation times of the implant site were measured from the preparation of the flap up to the insertion of the implant.

Immediately after the end of the surgical procedure, a questionnaire on the operative difficulty was compiled by the operator. In particular, the two techniques were compared considering two factors: the easiness in obtaining a correct axis of implant insertion and the quality of visibility.

Furthermore, a cumulative judgement was done for the whole procedure, as “simple,” “medium difficulty,” or “difficult.”

## 3. Statistical Analysis

SPSS software (SPSS Inc. Chicago, IL) was used for statistical analysis. A value of *p* < 0.05 was used in rejecting the null hypothesis.

In addition, continuous data were analyzed using nonparametric tests given the asymmetric distribution of some data sets.

The Friedman test was used to evaluate the significance of VAS differences between the groups over time. The Wilcoxon test was used to intercept differences between the groups at each time point. The Wilcoxon rank signed test was used as the post hoc test for pairwise comparison to evaluate the significance of VAS differences between the groups.

A Cochran test was used to assess the significance of differences in the frequency of intake of painkillers between the groups over time. Subsequently, a McNemar test was used for post hoc analysis and to assess the significance of differences in the frequency of intake of painkillers between groups each time point.

After having calculated the mean operative times for G1 and G2, the Mann–Whitney test was used to compare the differences in surgical times between D2 and D3 bone types within each group, while the Wilcoxon test was used to intercept the differences between the groups in bone types D2 and D3, respectively.

The Cochran test was used to test the differences in the operator questionnaire answers between the groups.

## 4. Results

From the analysis carried out on the comparison of the VAS scale of the statistical units treated with drill and piezoelectric methodology, a statistically significant difference was found in regard to the intraoperative symptomatology between each group over time (Friedman test: G1, *p* < 0.001; G2, *p* < 0.001). Differences of VAS values within the groups and between the groups at each time point are reported in Table 1.

**Figure 1:**
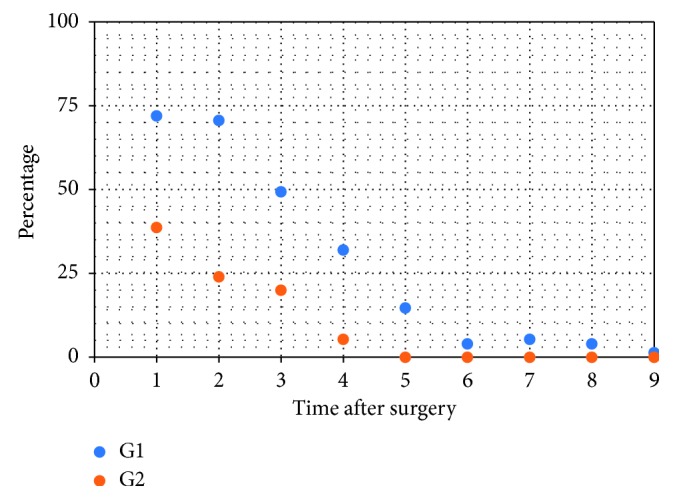
Frequency of intake of painkillers in percentage.

The differences in the frequency of intake of painkillers between the groups over time showed statistical significance (Cochran test; G1, *p* < 0.001; G2, *p* < 0.001) (Figure 1).

**Figure 2:**
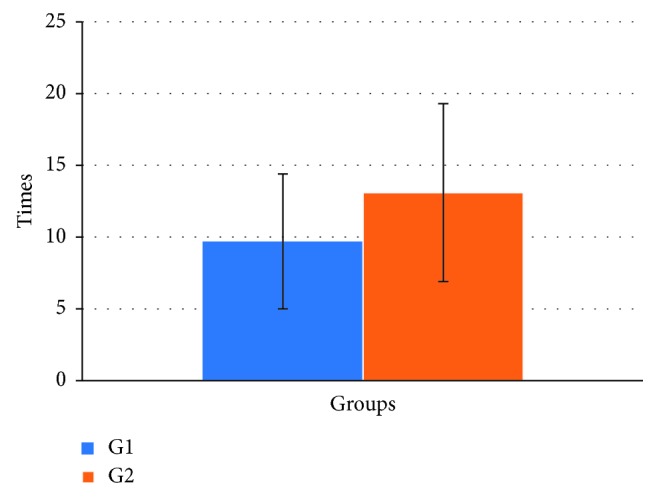
Operative surgery duration evaluated for the two study groups (min.). Significant difference with G1; Mann–Whitney test, *p* < 0.005.

From the analysis carried out on the comparison between the average surgical times of preparation of the implant sites in the experimental groups, it was found that the average time in the G1 was 9.7 minutes (±4.7), whereas in G2, it was 13.1 minutes (±6.2) (Figure 2).

Considering the different bone types, no differences were found in preparation times within the study groups (Mann–Whitney test, *p*=NS), whereas a significant difference was found between the groups, both for D2 and D3 densities (Wilcoxon test: D2, *p* < 0.05; D3, *p* < 0.001) (Table 2).

Answers given by the oral surgeon to the questionnaires showed a significant difference between G1 and G2 only for the quality of visibility (McNemar test, *p* < 0.05). Easiness in reaching the correct axis of insertion and the global judgement on the difficulty of the surgery did not show significant differences (Cochran test, *p* < NS).

## 5. Discussion

The objective of the present investigation was firstly to monitor postoperative pain of implants placed in sites prepared with drills and ultrasonic inserts.

The results of this clinical study showed significant effectiveness of the ultrasonic technique for performing implant preparation with reduction of pain.

The results also suggest that these benefits may be at the cost of increased operating time with the piezoelectric device. The findings therefore raise the possibility of improved clinical healing after osteotomy with the piezoelectric device compared with conventional rotary burs, and this is consistent with clinical and histological studies in rats [1, 18].

Also, the painkiller assumption showed a significant difference between G1 and G2. Until the fifth day after surgery, a higher use of painkillers was observed for G1. The reduced intake of painkillers in G2 allowed the patient to experience the implant insertion as a less invasive intervention.

However, the results of our study showed that the osteotomy performed with ultrasound extends the surgical times in respect to the drill preparation.

In the literature, it has been shown that piezoelectric osteotomy requires a longer intervention time than osteotomy with conventional drills [13, 15, 19–21]. As reported in some studies, piezoelectric bone surgery causes an acceleration of the healing processes at the level of the bone matrix, stimulating cell proliferation and its synthesis [22]. These advantages are the consequence of a more secure crestal osteotomy, as the ergonomic piezoelectric pivoting handpiece, without the initial “waving” phenomena typical of each rotating system, allows a more stable positioning of the guide insert on the crest profile, for the creation of the first implant hole, making the initial osteotomy less traumatic by exploiting the cavitational process with constant irrigation. All this, in biological terms, translates into a reduction of thermal stress on the bone tissue, maintenance of a better bone vitality, better compliance with osteoblastic turnover, and a possible respect of soft tissues and any noble anatomical structures (inferior alveolar nerve, Schneiderian membrane, etc.) contiguous to the osteotomy [13–15].

The prospect of using piezosurgery promises to revolutionize implantology, but the professional skill and training for its use should be taken into consideration because the technique requires a longer surgical time compared with the use of conventional rotary and oscillating saws. This occurs when deep cuts into the bone are necessary, and the system is less efficient. Although the cutting speed decreased, temperatures rose, so pauses were necessary to let the system cool down. In these cases, the combination of piezosurgery for the initial incision and a chisel for the final osteotomy of the bone was useful [23]. But the majority of studies agree that the piezoelectric device is extremely efficient and precise and recommend its use [24]. These observations were also supported by the present study; in fact, a lower pain was present for osteotomies performed by an ultrasonic device. The piezoelectric technique, in addition to being more tolerable, in terms of intraoperative comfort, and in the phases following surgery for the patient, has shown surgical advantages for the operator [25–27]. Although the operative times are higher for G2, they had no substantial influence on the global evaluation of the surgery. In fact, when the operator had to evaluate the difficulty of the implant insertion, no differences were found in comparison with G1 except for the visibility of the operative field that was better in G2. This is allowed by the cavitational effect of the piezoelectric device which allows for a clean and sterile operating field, ensuring the operator greater visibility, with the advantage of safer surgery.

Furthermore, comparative histological studies between piezoelectric devices, saws, and burs highlighted the superiority of the piezoelectric device in terms of protection of anatomic structures and, consequently, a better healing process. A remarkable feature of the piezoelectric device is its good manageability, which makes it easy for the surgeon to create a straight osteotomy line, without any learning period [28]. In this sense, a promising technical evolution is the combination of the intraoral navigation system with ultrasonic drilling to achieve a more safe and precise implant site preparation with a reduced invasiveness [29].

Within the limits of the present study, such as the choice of a single operator for the evaluation of the easiness of surgery or the choice of few parameters (VAS and painkillers assumption) for the evaluation of the patient's comfort, the results showed that piezosurgery was more comfortable in the implant phases and less painful in the postoperative one, bringing advantages in terms of acceptance of implant-supported prosthetic rehabilitation.

## 6. Conclusions

In recent years, the excellent results in the use of dental implants have greatly expanded the treatment options related to the replacement of missing teeth. Implantology has now reached levels of reliability and predictability of success over time that, associated with a high rate of safety, makes it a daily surgical practice.

The evolution of materials and techniques, with an increasing knowledge of the mechanism of healing processes, has also contributed to a reduction of numerous rehabilitative limitations. However, the success of a therapy is often determined only by the clinical result based on objective parameters, without taking into account the subjectivity of the patient and the operator.

The present study took these aspects into consideration, finding a positive response in implant surgery with the piezoelectric method compared to the traditional technique.

In light of the above, traditional surgery undoubtedly maintains the record for the speed of execution of the techniques. However, ultrasounds applied to the implant site preparation are a method of bone surgery that presents fewer risks related to operative maneuvers and greater comfort for the patient.

## Figures and Tables

**Table 1:** Pain rate of patients in intraoperative and postoperative times recorded by VAS Score.

Group	VAS										
	Intraop.	8 hours	1 day	2 days	3 days	4 days	5 days	6 days	7 days	15 days	Diff.^*∗*^
G1	4.05	3.58^a^	2.72^b^	2.28^c^	1.67^d^	0.86^e^	0.77	0.49^f^	0.33^g^	0.05^h^	*p*=0.000
G2	2.51	2.09	1.47^b^	1.02^j^	0.70^k^	0.42^l^	0.26	0.12	0.09	0.02	*p*=0.000
Diff.^*∗∗*^	*p*=0.000	*p*=0.000	*p*=0.000	*p*=0.000	*p*=0.000	*p*=0.002	*p*=0.001	*p*=0.007	*p*=0.014	*p*=0.317	

^*∗*^Friedman test; ^*∗∗*^Wilcoxon test. ^a^Significant difference with “intraop.” VAS; Wilcoxon test, *p* < 0.05. ^b^Significant difference with “8 hours” VAS; Wilcoxon test, *p* < 0.001. ^c^Significant difference with “1 day” VAS; Wilcoxon test, *p* < 0.05. ^d^Significant difference with “2 days” VAS; Wilcoxon test, *p* < 0.001. ^e^Significant difference with “3 days” VAS; Wilcoxon test, *p* < 0.001. ^f^Significant difference with “5 days” VAS; Wilcoxon test, *p* < 0.05. ^g^Significant difference with “6 days” VAS; Wilcoxon test, *p* < 0.05. ^h^Significant difference with “7 days” VAS; Wilcoxon test, *p* < 0.05. ^j^Significant difference with “1 day” VAS; Wilcoxon test, *p* < 0.001. ^k^Significant difference with “2 days” VAS; Wilcoxon test, *p* < 0.05. ^l^Significant difference with “3 days” VAS; Wilcoxon test, *p* < 0.05.

**Table 2:** Operative surgery duration evaluated considering the different bone types.

Groups	D2 mean times (min)	D3 mean times (min)	Diff.^*∗*^
G1	10.74 ± 5	8.6 ± 4.6	*p*=0.133
G2	13.04 ± 6.9	13.15 ± 5.5	*p*=0.516
Diff.^*∗∗*^	*p*=0.000	*p*=0.000	

^*∗*^Mann–Whitney test. ^*∗∗*^Wilcoxon test.

## Data Availability

All data used (pain rate of patients in intraoperative and postoperative times recorded by VAS Score) to support the findings of this study are available from the corresponding author upon request. We have annotated the entire data building process and empirical techniques presented in the paper.
